# Genome Sequence of *Proteus mirabilis* Clinical Isolate C05028

**DOI:** 10.1128/genomeA.00167-14

**Published:** 2014-03-27

**Authors:** Xiaolu Shi, Yuanfang Zhu, Yinghui Li, Min Jiang, Yiman Lin, Yaqun Qiu, Qiongcheng Chen, Yanting Yuan, Peixiang Ni, Qinghua Hu, Shenghe Huang

**Affiliations:** a Southern Medical University, Guangzhou, China; b Shenzhen Center for Disease Control and Prevention, Shenzhen, China; c BGI-Shenzhen, Shenzhen, People’s Republic of China

## Abstract

Genomic DNA of *Proteus mirabilis* C05028 was sequenced by an Illumina HiSeq platform and was assembled to 39 scaffolds with a total length of 3.8 Mb. Next, open reading frames (ORFs) were identified and were annotated by the KEGG, COG, and NR databases. Finally, we found special virulence factors only existing in *P. mirabilis* C05028.

## GENOME ANNOUNCEMENT

*Proteus mirabilis* is found in soil, water, and the human intestinal tract and is characterized by its swarming motility, ability to ferment maltose, and inability to ferment lactose (1). The most common infection occurs when *P. mirabilis* moves to the urethra and bladder. However, we isolated 27 *P. mirabilis* strains from stool samples of patients infected in the food-borne disease outbreak in 2005 in Shenzhen, Guangdong, China. Here, we report the whole-genome sequence of a new *P. mirabilis* strain (C05028) that was isolated from patients suffering from diarrhea in this outbreak.

The genomic DNA of *P. mirabilis* C05028 was sequenced by the Illumina HiSeq 2000 and was used to construct two libraries of 500 bp and 2 kb. A total of 133 million bp reads were generated, reaching a depth of ~350-fold genome coverage, and were assembled into 39 scaffolds (≥200 bp in size), with a total length of 3,817,619 bp and containing 24,529-bp gap regions.

Open reading frames (ORFs) were identified with Glimmer version 3.0 (2), and 3,475 protein-coding sequences (CDSs) were predicted, with an average gene length of 928 bp. Repeat regions were predicted, including transposon sequences and tandem repeat sequences using RepeatMasker (3), RepeatProteinMasker, and the TRF software. Finally, we found 5.6-kb different transposable element (TE)-related sequences, consisting of 0.15% of the genome. The gene functions were annotated into the KEGG (4), COG (5), Swiss-Prot, TrEMBL (6), and NR databases using BLASTp. Homologous proteins were identified by BLASTp, with the criteria of an *E* value cutoff of 1e - 5 and a minimum aligned sequence length coverage of 50% of a query sequence. Using the above criteria yielded 2,595 protein families, with 2,582 single-copy protein families.

Our ultimate goal was to find special virulence factors of *P. mirabilis* C05028 by comparison with other nonpathogenic bacteria. We identified about 32,000 single-nucleotide polymorphisms (SNPs) using the MUMmer tool (7) and found that some SNPs were located in predicted genes of *P. mirabilis* C05028 related to virulence factors. Meanwhile, we found several virulence factors existing only in strain *P. mirabilis* C05028.

### Nucleotide sequence accession number.

This whole genome of *P. mirabilis* C05028 has been deposited at DDBJ/EMBL/GenBank under the accession no. ANBT00000000. The version described in this paper is the ﬁrst version.
